# Radiotherapy for inoperable and refractory endometriosis presenting with massive hemorrhage: a case report

**DOI:** 10.1186/1752-1947-6-308

**Published:** 2012-09-18

**Authors:** Takuma Nomiya, Mayumi Harada, Hiroko Sudo, Ibuki Ota, Mayumi Ichikawa, Motohisa Suzuki, Misako Murakami, Kenji Nemoto

**Affiliations:** 1 Department of Radiation Oncology, Yamagata University Hospital, 2-2-2, Iida-Nishi, Yamagata City, Yamagata, 990-9585, Japan

**Keywords:** Endometriosis, Radiotherapy, Menopause

## Abstract

**Introduction:**

Many patients with endometriosis are treated with medication or by surgical approaches. However, a small number of patients do not respond to medication and are inoperable because of comorbidities. This case report shows the effectiveness of radiotherapy for refractory endometriosis and includes a time series of serum estradiol levels.

**Case presentation:**

A 47-year-old Asian woman presented to our facility with uncontrolled endometriosis refractory to medication. Our patient was considered inoperable because of severe idiopathic thrombocytopenic purpura, and underwent radiotherapy for massive genital bleeding requiring blood transfusions. A radiation dose of 20Gy in 10 fractions was delivered to the pelvis, including the bilateral ovaries, uterus, and myomas. An additional 10Gy in five fractions was delivered to the endometrium to control residual bleeding. Genital bleeding was completely inhibited on day 46 after radiotherapy. Hormonal analysis revealed that radiotherapy induced post-menopausal status. Two years after radiotherapy, atypical genital bleeding had not recurred and has been well controlled without side effects.

**Conclusions:**

Disrupted ovarian function is an adverse effect of radiotherapy. However, radiotherapy can be useful for inducing menopause. In cases of medication-refractory or inoperable endometriosis, radiotherapy would be an effective treatment option.

## Introduction

Endometriosis is a gynecological condition characterized by extra-uterine endometrial-like cells, which often proliferate and cause hematomas, menstrual pain, or other symptoms, in conjunction with hormonal changes.

In general, pain relievers and hormonal supplements are prescribed during menopause [1-6]. However, when endometriosis is refractory to medicinal treatments, surgical intervention is required, including endometrial tissue debridement or hysterectomy with/without ovarian excision [7-10].

In this study, we report the case of a patient with refractory and inoperable endometriosis treated with radiotherapy.

## Case presentation

A 47-year-old Asian woman presented to the Department of Radiation Oncology, Yamagata University Hospital, with a chief issue of massive atypical genital bleeding.

Our patient was diagnosed as having idiopathic thrombocytopenic purpura (ITP) at three years of age; however, splenectomy, steroid therapy, and γ-globulin therapy failed to improve her condition. She had severe thrombocytopenia (platelet count <10,000 cells/mm^3^) at the time of radiotherapy. Because of her history of ITP, surgical intervention for endometriosis was ruled out.

**Figure 1:**
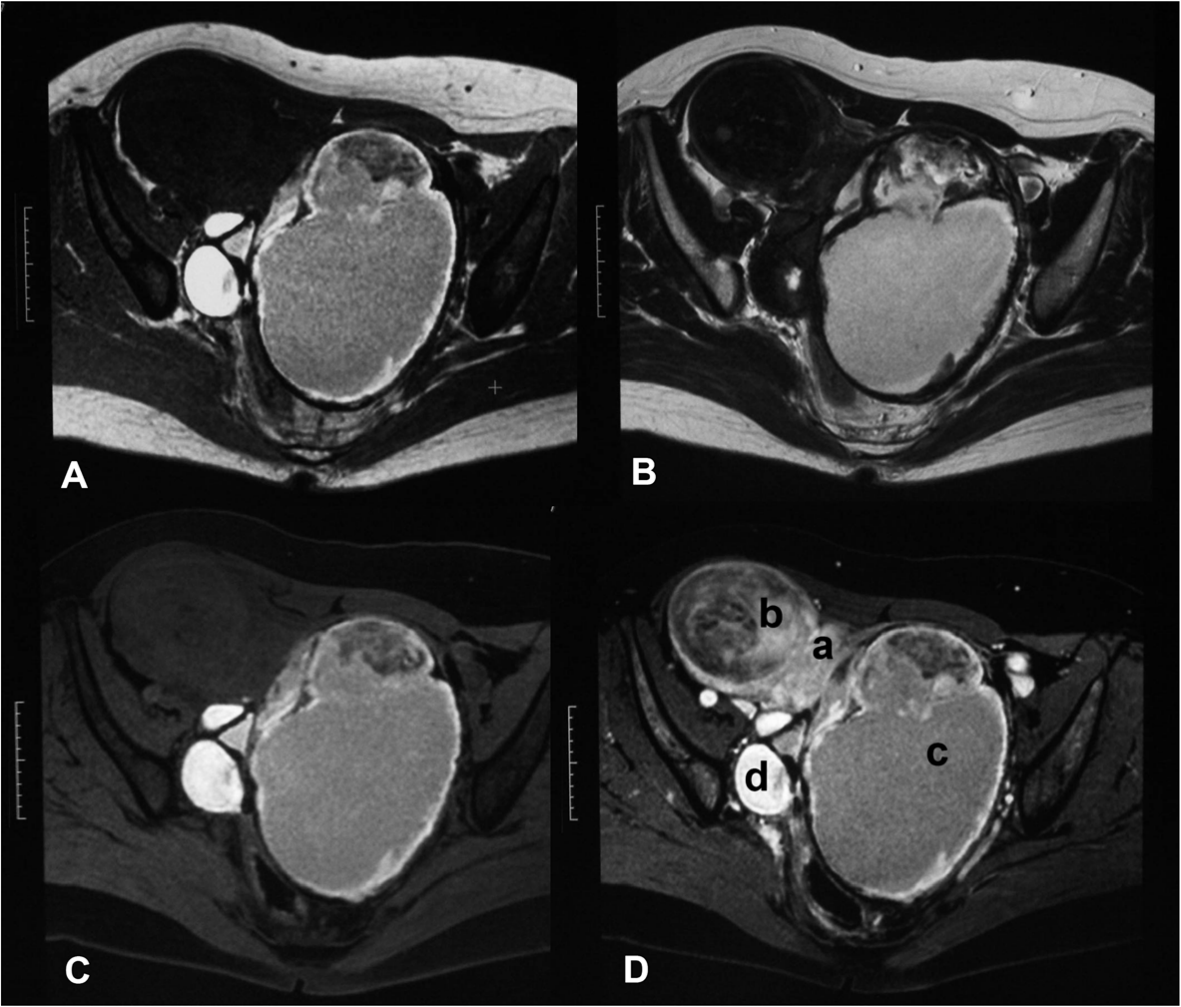
**Pelvic magnetic resonance images of our patient before radiotherapy. (A)** T1-weighted image; (**B**) T2-weighted image; (**C**) fat-saturated T1-weighted image; (**D**) fat-saturated contrast-enhanced T1-weighted image. (**a**) Normal uterine body; (**b**) subserous myoma uteri; (**c**) huge chocolate cysts (endometrial cysts) arising from the left ovary; (**d**) endometrial cysts arising from the right ovary.

Our patient had been experiencing atypical genital bleeding for 10 years. She was diagnosed as having bilateral ovarian chocolate cysts at the age of 40. Although hormonal therapies (luteinizing-hormone-releasing hormone (LH-RH) analog and contraceptive pills) were administered, they were not effective, and medicinal treatments were suspended without controlling the symptoms. Our patient experienced massive genital bleeding requiring daily transfusions of four to six units of packed red blood cells (PRBCs) before radiotherapy (Figure 1 shows the magnetic resonance images at that time).

Bilateral ovaries (endometriotic cysts) were included as radiotherapy targets to obtain radiation-induced menopause. The uterine myoma and normal endometrium were also included as targets as they may have been the origins of genital bleeding. Radiotherapy of 20Gy in 10 fractions with 10MV photons was delivered to the targets, which was sufficient for inducing menopause. To correct residual genital bleeding as per our patient's wishes, another 10Gy radiation in five fractions was provided to the normal endometrium.

**Figure 2:**
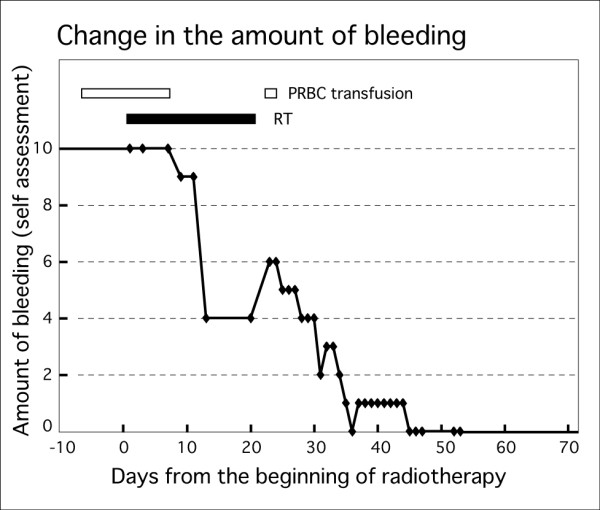
**Time course and amount of genital bleeding.** The amount of genital bleeding was scored by self-assessment from 0 (no bleeding) to 10 (the worst ever experienced). Before radiotherapy, our patient experienced massive bleeding, requiring daily transfusion of two to six units of packed red blood cells. The amount of genital bleeding had reduced to half of the initial amount at the end of radiotherapy. Thereafter, the amount of bleeding continued to reduce and was completely inhibited by day 46. PRBC; packed red blood cells, RT; radiotherapy.

Figure 2 shows the time course and blood volumes of the atypical genital bleeding. The first day of radiotherapy was set as day 1, and the amount of bleeding per day was assessed as often as possible. Because it is very difficult to measure a precise volume of blood loss, a numerical rating scale (NRS) from 0 (no bleeding) to 10 (excessive bleeding) was used along with self-assessment. Decreased bleeding occurred by day 14, and complete arrest of bleeding was achieved by day 46. Following treatment, no genital bleeding has been reported, and no blood transfusion has been performed for two years after radiotherapy.

**Figure 3:**
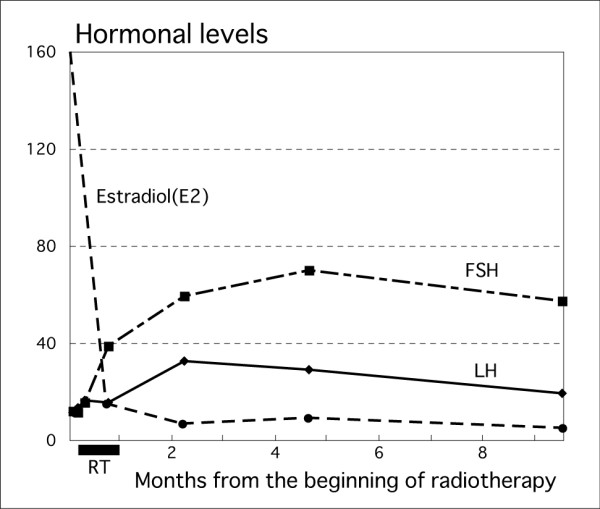
**Changes in serum hormonal levels from the onset of radiotherapy.** The serum estradiol level, which was very high before radiotherapy, rapidly decreased after the onset of radiotherapy (dashed line; pg/mL). The serum luteinizing hormone level (solid line; mIU/mL) and serum follicle stimulating hormone level (long dashed, short dashed line; mIU/mL) gradually increased after radiotherapy. These changes in hormonal levels suggested that our patient was of post-menopausal status. FSH; follicle stimulating hormone, LH; luteinizing hormone, RT; radiotherapy.

Figure 3 shows the changes in serum hormone levels. Before radiotherapy, her serum estradiol (E2) level was 161pg/mL (normal range; follicular phase: 19 to 226, menopause: <39), serum follicle stimulating hormone (FSH) level was 12.2mIU/mL (normal range; follicular phase: 4 to 12, menopause: 39 to 176), and serum luteinizing hormone (LH) level was 11.7mIU/mL (normal range; follicular phase: 1.1 to 8.1, menopause: 11 to 68.6). The serum E2 level rapidly decreased after the start of radiotherapy and reached the post-menopausal level two months later. At four months after radiotherapy, the serum FSH level increased to 69.7mIU/mL (post-menopausal level) and the serum LH level increased to 29.2mIU/mL (post-menopausal level). These serum hormone levels suggested that radiation-induced menopause was successfully obtained two months after the start of radiotherapy.

## Discussion

Hormone therapies for endometriosis include medroxyprogesterone, danazol, and gonadotropin-releasing hormone (GnRH), among other drugs [1-3]. These drugs have favorable effects for symptoms of endometriosis, but they occasionally cause side effects such as spotting, weight gain, skin changes, and hypo-estrogenism [4-6].

However, laparoscopic stripping of endometriomas is reported to be as effective as surgical treatments [7]. Laparoscopic excision of the cyst wall of the endometrioma reduces the recurrence rates of endometriomas, dysmenorrhea symptoms, non-menstrual pelvic pain, and requirement for further surgery [8].

In our patient’s case, surgery could not be considered as an option because our patient had underlying thrombocytopenia (platelet count <10,000 cells/mm^3^) due to ITP. Our patient seemed to have a very complicated multiple disease scenario, with: (i) endometriosis (bilateral ovaries), (ii) secondary chocolate cysts, (iii) adenomyosis (complicating about 10% of the endometriosis), (iv) thrombocytopenia due to ITP and (v) uterine myoma (subserous myoma). As it was considered that all of the diseases might be a cause of bleeding, a particular state that ‘atypical bleeding gets worse according to female hormone levels’ seemed to be mainly caused by endometriosis. Therefore treatment was performed so as to control the excess of female hormones. Treatment to control the bleeding with interventional radiology procedures was also considered, however, interventional radiology procedures were not indicated because they would not improve the essential cause of disease, there was uncertainty of their effect and there was an inherent risk associated with invasive procedures. Although oral contraceptives and a GnRH analog were administered, both drugs failed to suppress the endometriosis, and only symptomatic treatments such as blood transfusion were performed. Ectopic endometrial-like cell activity is dependent on female hormone levels. Therefore, anti-estrogen, androgen, and GnRH analog therapies were implemented for symptomatic relief of the endometriosis.

In our patient’s case, estrogen secretion was successfully inhibited by bilateral ovarian irradiation. A previous study estimated that the 50% lethal dose (LD_50_) for human oocytes was 4Gy or less [11]. Several studies reported the ability of radiotherapy for ablating remnant ovarian tissue in recurrent or refractory endometriosis, based on the limited tolerance of human oocytes to radiation damage [12-14]. These studies reported that menopause was induced at a dose of 15 to 30Gy radiation. A previous clinical study reported that menopausal status had been obtained in almost all patients with breast cancer with distant metastases using a radiation dose of 10Gy in four fractions [15].

Radiotherapy is a less invasive treatment and is widely applicable regardless of a patient’s age or operability. Therefore, radiotherapy should be considered for patients with medication-refractory and/or inoperable endometriosis. However, one limitation of radiotherapy is the irreversible loss of ovarian function; consequently, careful consideration should be given to adolescents and younger women who desire pregnancy. In our patient’s case, radiotherapy was administered because our patient did not desire ovarian preservation and because she was at risk of life-threatening blood loss.

## Conclusions

We have demonstrated a case of refractory endometriosis successfully treated with radiotherapy and conclude that radiotherapy is an effective treatment option for patients with medication-refractory or inoperable endometriosis.

## Consent

Written informed consent was obtained from the patient for publication of this case report and accompanying images. A copy of the written consent is available for review by the Editor-in-Chief of this journal.

## Competing interests

The authors declare that they have no competing interests.

## Authors’ contributions

TN analyzed and interpreted the data from our patient and prepared the manuscript. KN, MM, and MS participated in the clinical work. MI, IO, HS, and MH analyzed and reviewed the data from our patient. KN and TN determined the clinical work. All authors have read and approved the final manuscript.
